# The day-to-day reliability of peak fat oxidation and FAT_MAX_

**DOI:** 10.1007/s00421-020-04397-3

**Published:** 2020-06-01

**Authors:** Oliver J. Chrzanowski-Smith, Robert M. Edinburgh, Mark P. Thomas, Nicos Haralabidis, Sean Williams, James A. Betts, Javier T. Gonzalez

**Affiliations:** Department for Health, University of Bath, Bath, BA2 7AY UK

**Keywords:** Peak fat oxidation, FAT_MAX_, Reliability, Exercise metabolism, Variation, Indirect calorimetry

## Abstract

**Purpose:**

Prior studies exploring the reliability of peak fat oxidation (PFO) and the intensity that elicits PFO (FAT_MAX_) are often limited by small samples. This study characterised the reliability of PFO and FAT_MAX_ in a large cohort of healthy men and women.

**Methods:**

Ninety-nine adults [49 women; age: 35 (11) years; $\dot{V}$O_2_peak: 42.2 (10.3) mL·kg BM^−1^·min^−1^; mean (SD)] completed two identical exercise tests (7–28 days apart) to determine PFO (g·min^−1^) and FAT_MAX_ (%$\dot{V}$O_2_peak) by indirect calorimetry. Systematic bias and the absolute and relative reliability of PFO and FAT_MAX_ were explored in the whole sample and sub-categories of: cardiorespiratory fitness, biological sex, objectively measured physical activity levels, fat mass index (derived by dual-energy X-ray absorptiometry) and menstrual cycle status.

**Results:**

No systematic bias in PFO or FAT_MAX_ was found between exercise tests in the entire sample (− 0.01 g·min^−1^ and 0%$\dot{V}$O_2_peak, respectively;* p* > 0.05). Absolute reliability was poor [within-subject coefficient of variation: 21% and 26%; typical errors: ± 0.06 g·min^−1^ and × / ÷ 1.26%$\dot{V}$O_2_peak; 95% limits of agreement: ± 0.17 g·min^−1^ and × / ÷ 1.90%$\dot{V}$O_2_peak, respectively), despite high (*r* = 0.75) and moderate (*r* = 0.45) relative reliability for PFO and FAT_MAX,_ respectively. These findings were consistent across all sub-groups.

**Conclusion:**

Repeated assessments are required to more accurately determine PFO and FAT_MAX_.

**Electronic supplementary material:**

The online version of this article (10.1007/s00421-020-04397-3) contains supplementary material, which is available to authorized users.

## Introduction

Considerable interest has grown in the concept of peak (or maximal) fat oxidation (PFO; a whole-body measure of the ‘maximal’ capacity to oxidise fat) and the exercise intensity that elicits PFO (i.e. FAT_MAX_) (Amaro-Gahete et al. 2019; Maunder et al. 2018). However, knowledge on the reproducibility of these parameters is crucial to be able to appropriately interpret the importance of PFO and FAT_MAX_ in the context of weight management (Dandanell et al. 2017a, b), metabolic health (Robinson et al. 2015) and/or endurance exercise performance (Frandsen et al. 2017).

Several studies have now investigated the day-to-day reliability (otherwise known as reproducibility, intra-individual variation or within-subject variation) of PFO and FAT_MAX_ across a range of exercise modes [e.g. treadmill (De Souza Silveira et al. 2016; Marzouki et al. 2014), cycle ergometry (Croci et al. 2014; Dandanell et al. 2017a, b) and ski ergometry (Hansen et al. 2019)] and populations [e.g. trained or recreationally trained athletes (Croci et al. 2014; De Souza Silveira et al. 2016) and individuals with low levels of cardiorespiratory fitness (Chrzanowski-Smith et al. 2018; Dandanell et al. 2017a, b)]. Notably, in some studies, large intra-individual variation has been reported. For example, Croci et al. (2014) compared the day-to-day reliability of three data analysis approaches to determine PFO and FAT_MAX_ [measured values (MV), fitting a third-order polynomial curve (P3) and the SINE model (SIN)] in fifteen moderately trained men and reported large 95% limits of agreements (95% LoAs; range ± 0.24–0.26 g·min^−1^ and 27–32% $\dot{V}$O_2_peak) and within-subject coefficients of variation (CVs; > 15%) across all approaches. Additionally, similarly large 95% LoA for PFO (± 0.13–0.15 g·min^−1^) has been reported in individuals with low levels of cardiorespiratory fitness (Chrzanowski-Smith et al. 2018; Dandanell et al. 2017a, b). However, others report lower CVs (< 10%) and 95% LoA (± ~ 0.10 g·min^−1^ and 8%$\dot{V}$O_2_peak) for PFO and FAT_MAX_, respectively (De Souza Silveira et al. 2016; Hansen et al. 2019; Marzouki et al. 2014).

A range of different methods (e.g. gas analysis systems, FAT_MAX_ protocols, data analysis approaches applied) have been employed to assess PFO and FAT_MAX_ that may partly account for such discrepancies in the day-to-day reliability values reported (Amaro-Gahete et al. 2019). Moreover, all prior reliability studies have been conducted in relatively small (*n* < 23) and homogenous samples. Similarly, the only prior study to explore the level of agreement between different data analysis approaches to determine PFO and FAT_MAX_ recruited thirty-two young, healthy adults (Chenevière et al. 2009). To date, a direct assessment of the day-to-day reliability of PFO and FAT_MAX_ across specific sub-populations employing a standardised methodology is yet to be explored but would greatly help to extend the generalisability of prior findings to wider populations.

Therefore, the main aims of this study were to: (1) explore the day-to-day reliability of PFO and FAT_MAX_ in a large sample of healthy men and women with varying levels of cardiorespiratory fitness, physical activity levels and body composition; (2) investigate whether the day-to-day reliability of PFO and FAT_MAX_ is similar across data analysis approaches and sub-populations; and (3) assess the level of agreement between different data analysis approaches [MV, fitting a least squares second-order polynomial curve (P2) and SIN] for determining PFO and FAT_MAX_. The hypotheses were that (1) large day-to-day variation would be evident for both PFO and FAT_MAX_ and (2) this would be consistent across data analysis approaches and sub-populations, alongside (3) higher levels of agreement between P2 and SIN compared to MV.

## Materials and methods

### Study design

This study was a cross-sectional study that involved three visits to the University of Bath, UK. All participants provided written informed consent prior to participating in the study. The study was performed in accordance with the Declaration of Helsinki and was approved by the Research Ethics Approval Committee for Health at the University of Bath (REF: EP 16/17 141) and the South West-Bristol NHS Research Ethics Committee (17/SW/0269) and registered on ClinicalTrials.gov: NCT03029364.

Briefly, participants completed two matched trial days (Trial A and Trial B) separated by 7–28 days that involved the assessment of anthropometrics, resting metabolic rate, a fasting venous blood sample and a FAT_MAX_ test. A third visit (Trial C) was also organised 2–7 days after Trial B that involved a dual-energy X-ray absorptiometry (DEXA) scan to assess body composition. Trials were completed after an overnight fast (10–12 h) and started at a similar time (± 1 h within participant) of the day (0630–1230 h). Over the 48-h preceding each trial, participants were asked to: (a) abstain from alcohol and strenuous physical activity; and (b) wear a physical activity monitor and replicate their dietary intake and physical activity (all confirmed by verbal questioning). Additionally, over the 7 days before Trial A, participants recorded a self-weighed diet diary and wore a physical activity monitor. On the morning of each trial, participants minimised physical activity and consumed 568 mL of water upon waking (see accompanying open access readme file for study protocol deviances (Chrzanowski-Smith et al. 2020). Participants also maintained their habitual lifestyle throughout their involvement in the study. All trials (within-subject) were performed under similar laboratory conditions [particularly for ambient temperature (CV = 4%) and barometric pressure (CV = 1%) with more variance in humidity (CV = 16%); *p* values for systematic differences between Trial A and Trial B > 0.187] where ad libitum water intake and use of fans were permitted.

### Participants

Ninety-nine healthy male and female adults (aged 18–65 years) were recruited from the South West region of the UK. Exclusion criteria included; age < 18 or > 65 years; having current or any history of cardio-pulmonary, metabolic or musculoskeletal disease; breastfeeding or was/potentially pregnant; a body mass index outside of < 18.5 and > 35 kg·m^−2^; not willing to meet the demands of the study or maintain their habitual lifestyle during their involvement; not being weight stable (± 5% body mass; self-reported) for at least the 3 months prior to their involvement; or any conditions or concurrent behaviour (including medication) that may have posed undue personal risk to the participant or introduced bias to the study. Participant characteristics are presented in Tables 1 and 2. In female participants who were eumenorrheic and not on contraceptive medication, trials were scheduled (based on self-reported and predicted phases) to take place in the same phase of the menstrual cycle. The menstrual cycle was split into two broad phases: the follicular and the luteal (which included ovulation). The success in controlling for menstrual cycle phase between Trial A and Trial B (based on self-report and predicted phases) was then objectively verified by the analysis of oestradiol and progesterone concentrations. As oestradiol concentrations can vary widely across the menstrual cycle, the follicular and luteal phases were determined by a progesterone concentration of < and ≥ 5 nmol·L^−1^, respectively (Oosthuyse et al. 2005). As shown in Supplementary Table 1, the success of controlling for menstrual cycle phase was varied. In all females whose menstrual cycle phase was matched between Trial A and Trial B (i.e. were tested in the same phase), testing occurred in the follicular phase (a progesterone concentration of < 5 nmol·L^−1^). If Trial A and Trial B occurred in a different phase of the menstrual cycle, participants were classed as non-matched. Female participants for whom it was unknown what phase of the menstrual cycle Trial A and/or Trial B occurred in (e.g. progesterone concentrations were not available) were grouped as ‘unknown’. Female participants who self-reported the absence of menstrual cycle for ≥ 365 days were classified as post-menopausal, where low concentrations of oestradiol and progesterone were apparent (Supplementary Table 1). Contraceptive use was categorised into four sub-groups: combined pill, progesterone-only pill, intrarauterine system (IUS) or intrauterine device (IUD).

**Table 1:** Participant demographic and lifestyle characteristics

	Total sample (*n* = 99)	Range	Male (*n* = 50)	Female (*n* = 49)
Age (years)	35 (11)	19–63	37 (39)^†^	33 (44)^†^
Ethnicity (% Caucasian)	90	–	96	83
Body stature (cm)^‡^	173.4 (8.6)	157.3–191.9	179.6 (6.5)	167.0 (5.1)
Body mass (kg)^‡^	71.2 (11.8)	48.0–106.2	79.5 (9.4)	62.8 (7.3)
BMI (kg·m^−2^)^‡^	23.6 (2.8)	18.5–32.9	24.7 (2.8)	22.5 (2.4)
Healthy (*n* = 70, 30 and 40)	21.9 (2.2)	18.5–24.7	23.1 (4.8)	21.6 (5.9)
Overweight (*n* = 27, 18, 9)	26.6 (4.9)	25.0–29.9	26.9 (4.9)	26.3 (3.5)
Obese (*n* = 2, 2 and 0)	31.8 (2.2)	30.7–32.9	31.8 (2.2)	–
Body fat % **	22.5 (7.9)	7.7–40.1	17.4 (5.6)	27.7 (6.3)
Fat mass (kg)	15.8 (5.6)	5.7–28.8	14.0 (5.5)	17.6 (5.2)
Fat mass index (kg·m^−2^)^‡‡^	–	1.62–10.9	4.5 (8.0)^†^	6.1 (7.7)^†^
Fat deficient (*n;* 12 and 15)	27	–	2.3 (1.3)	4.3 (1.7)
Healthy (*n;* 30 and 29)	59	–	4.6 (2.6)	6.6 (3.8)
Excess adiposity (*n;* 7 and 5)	12	–	6.8 (2.2)	10.5 (1.5)
Obese (*n*; 1)	1	–	–	–
Fat-free mass (kg)	54.5 (43.5)^†^	36.4–79.9	64.3 (27.9)^†^	44.9 (24.6)^†^
Fat-free mass index (kg·m^−2^)^‡‡‡^	18.24 (2.6)	13.3–24.6	20.3 (1.8)	16.2 (1.27)
Waist–hip circumference^‡‡^	–	0.67–0.99	0.82 (0.13)	.75 (0.05)
Energy intake (kcal·day^−1^)	2365 (625)	1235–4852	2724 (588)	1999 (416)
Carbohydrates (g·day^−1^)	245 (94)	37–537	285 (86)	204 (73)
Fat (g·day^−1^)	96 (35)	38–212	109 (31)	84 (35)
Protein (g·day^−1^)	105 (34)	43–221	120 (34)	89 (27)
Alcohol (g·day^−1^)	4^†^	0–54	5 (54)^†^	0 (24)^†^
Physical activity level (*n* = 96, 50, 46)	1.72^†^	1.35–2.42	1.87 (1.37–2.42)^†^	1.57 (1.35–2.22)^†^
Sedentary (*n* = 3, 1, 2)	1.37^†^	1.35–1.39	–	1.37 (1.35–1.39)^†^
Low active (*n* = 29, 5, 24)	1.52^†^	1.40–1.59	1.53 (1.43–1.59)^†^	1.52 (1.40–1.58)^†^
Moderately active (*n* = 39, 21, 18)	1.74^†^	1.60–1.87	1.74 (1.61–1.87)^†^	1.65 (1.60–1.87)^†^
Very active (*n* = 25, 23, 2)	2.03^†^	1.91–2.42	2.03 (1.91–2.42)^†^	2.08 (1.93–2.22)^†^

Data presented as mean (SD) unless otherwise stated

^†^Median (range)

^‡^Average of Trial A and B

^‡‡^Derived from Trial C

^‡‡‡^Average of Trial A and B using DEXA BF% from Trial C

*BMI* = body mass index; Fat Mass Index classification derived from Kelly et al. (2009). Physical activity level categories derived from Brooks et al. (2004). No whole sample average data reported for fat mass index and waist–hip circumference, nor fat mass index classifications due to different male and female thresholds. When *n* = 1 in a sub-group data not reported

**Table 2:** Participant metabolic characteristics and metabolite and hormone concentrations

	Whole sample	Range	Male	Female
$\dot{V}$O_2_peak (L·min^−1^; *n* = 98, 50, 48)	3.0 (0.90)	1.6–5.4	3.7 (0.6)	2.3 (0.4)***
$\dot{V}$O_2_peak (mL·kg BM^−1^·min^−1^*)*	42.2 (10.3)	22.3–65.7	47.3 (9.8)	36.8 (7.8)***
$\dot{V}$O_2_peak (mL·kg FFM^−1^·min^−1^)	54.0 (9.6)	33.6–73.0	57.1 (9.8)	50.8 (8.2)***
Peak power output (W; *n* = 97, 50, 47)	230	105–434	295 (303)	155 (198)***
HR_MAX_, (beats·min^−1^; *n* = 88, 43, 45)	180 (10)	148–204	179 (11)	181 (10)
PFO (g·min^−1^, *n* = 97, 50, 47)	0.31 (0.11)	0.10–0.74	0.35 (0.12)	0.28 (0.09)***
PFO (mg·kg BM^−1^·min^−1^)	4.45 (1.65)	1.55–11.30	4.44 (1.68)	4.46 (1.63)
PFO (mg·kg FFM^−1^·min^−1^)	5.72 (1.94)	2.17–13.89	5.34 (1.83)	6.13 (1.98)*
FAT_MAX_^a^ (%$\dot{V}$O_2_peak)	39 (10)	21–65	38 (12)	40 (9)
NEFA (mmol·L^−1^; *n* = 79, 37, 42)	0.35^†^	0.08–1.05	0.31 (0.87)^†^	0.42 (0.90)^†^ *
Triglyceride (mmol·L^−1^; *n* = 79, 37, 42)	0.64^†^	0.36–1.51	0.67 (1.15)^†^	0.63 (0.76)^†^
Glucose (mmol·L^−1^; *n* = 79, 37, 42)	5.56 (0.45)	4.39–6.83	5.72 (0.46)	5.37 (0.35)***
Lactate (mmol·L^−1^; *n* = 79, 37, 42)	0.70^†^	0.43–1.61	0.74 (1.16)^†^	0.59 (0.60)^†^ **
Insulin (pmol·L^−1^;*n* = 79, 37, 42)	22.14 (5.39)	11.82–40.31	22.83 (4.92)	21.35 (5.85)
Oestradiol (mmol·L^−1^; *n* = 78, 41, 36)	83.7^†^	18.4–1845.0	68.14 (235.7)^†^	266.8 (1826.6)^†^ ***
Progesterone (nmol·L^−1^; *n* = 78, 41, 36)	0.68^†^	0.22–37.25	0.61 (1.15)^†^	0.79 (36.98) ^†^ **

Data presented as mean (± SD) unless otherwise stated below

^†^Median (range); $\dot{V}$O_2_peak = peak oxygen consumption, included in total *n* is *n* = 1 estimated on Trial A and B by Astrand-Rhyming Nomogram (Astrand and Ryhming 1954) and *n* = 1 excluded as only completed Trial A; Peak Power Output, *n* = 2 excluded as stopped prior to exhaustion (*n* = 1) and did not complete Trial B (*n* = 1); HR_MAX_ = maximum recorded heart rate, *n* = 88 due to issues with the heart rate monitor; PFO and FAT_MAX_, measured values approach, *n* = 2 excluded as no metabolic data was available due to hyperventilation in both trials (*n* = 1) and did not complete Trial B (*n* = 1); Metabolites and hormones measured in plasma; NEFA = non-esterified fatty acids; *p < .05, female vs male; *p < 0.05; **p < .01, female vs male; ***p ≤ .001, female *vs* male

### Anthropometrics

Anthropometric measurements were performed upon participant arrival at the laboratory. Body stature was measured to the nearest 0.1 cm using a wall-mounted stadiometer (Holtain Ltd, Pembrokeshire, UK) alongside body mass to the nearest 0.1 kg using electronic weighing scales (BC-543 Monitor, Tanita, Tokyo, Japan). During Trial C, body stature and body mass were assessed in addition to waist and hip circumference [to the nearest 0.1 cm using a non-elastic measuring tape (SECA 201, Hamburg, Germany)] and a whole-body dual-energy X-ray absorptiometry scan was taken to quantify fat and fat-free mass (Discovery, Hologic, Bedford, UK).

### Blood sample and analysis

After resting metabolic rate was assessed, a 10-mL whole venous blood sample was obtained from an antecubital vein (BD Vacutainer Safety Lok, BD, USA). Blood samples were equally dispensed into either a 5-mL ethylenediaminetetraacetic acid-coated tube (K3 EDTA, Sarstedt, Germany) or a 10-mL serum/clotting activator tube (Serum Z/10 mL, Sarstedt, Germany) for plasma and serum separation, respectively. Samples for plasma were immediately centrifuged (1700*g* for 15 min at 4 °C); whereas, serum tubes were left to clot for 20–30 min at room temperature prior to centrifugation (standardised within-participant; Heraeus Biofuge Primo R, Kendro Laboratory Products Plc., UK). The plasma and serum samples, alongside the buffy coat layer from the K3 EDTA tube, were dispensed equally into 0.5-mL aliquots and immediately frozen at − 20 °C, before longer-term storage at − 80 °C for later batch analysis. The plasma samples were analysed for concentrations of various metabolites and hormones according to manufacturer instructions. Total plasma non-esterified fatty acids (NEFA; Cat No: FA115; intra-assay < 5% and inter-assay < 5%), glucose (Cat No: GL3815; < 5% and < 6%), lactate (Cat No: LC3980; < 4% and < 5%) and triglycerides (Cat No: TR3823 < 4% and < 4%) concentrations were run in singular on a Daytona Rx Series (Randox Laboratories, Crumlin, NI, USA). Total 17β-oestradiol (Elecsys Estradiol III; < 7% and < 11%) and progesterone (Progesterone III; < 11% and < 23%) concentrations were run in singular on a Cobas 8000 (Modular analytics Cobas e 602, Roche Diagnostics, Rotkreuz, Switzerland). Total plasma insulin concentrations were analysed by an enzyme-linked immunosorbent assay (ELISA) kit in duplicate (Cat No: 900095, Cyrstal Chem, Illinois, USA) with absorption determined by a microplate reader (SPECTROstar Nano, BMG LABTECH, Ortenberg, Germany) at wavelengths specified by the manufacturer (intra-assay CV < 2%; inter-assay CV < 24%).

### FAT_MAX_ test

After resting metabolic rate was assessed and a fasting venous blood sample was obtained, participants then completed a FAT_MAX_ test. This test adopted a protocol previously validated in individuals who were trained (Achten et al. 2002) and in individuals who had low cardiorespiratory fitness (Chrzanowski-Smith et al. 2018). Briefly, the FAT_MAX_ test was an incremental graded cycling test to volitional exhaustion completed on a mechanically braked cycle ergometer (Monark Peak Bike Ergomedic 894E, Varberg, Sweden). The graded test comprised of four-min stages for the first seven stages and two-min stages from the eighth stage onwards. The initial power output was ~ 30 or 40 W and increased by ~ 25 W (excluding the 10-W increment between first and second stages in the 30-W protocol) over the next five and six stages, respectively, and by ~ 50 W from stage seven onwards. One-min expired gas samples, heart rate and RPE were collected in the final min of the first seven stages and upon the participant’s signal of one-min remaining before volitional exhaustion. The graded test was used to determine:

- Peak fat oxidation (g·min^−1^);
- FAT_MAX_ (expressed as a % of $\dot{V}$O_2_peak);
- Peak power output (W; power output of the last completed stage, plus the fraction of time in the final non-completed stage, multiplied by the Watt increment of that stage);
- An estimate of peak oxygen consumption ($\dot{V}$O_2_peak; mL·kg^−1^·min^−1^)

Three data analysis approaches were applied to determine PFO and FAT_MAX._ These involved: (1) the measured values approach [MV; the stage with the highest recorded fat oxidation value and the corresponding $\dot{V}$O_2_ (Achten et al. 2002)]; (2) the fitting of a least squares second-order polynomial curve to the measured fat oxidation rates (P2) (Hansen et al. 2019; Stisen et al. 2006); and (3) the Sine model [SIN; a mathematical model that applies a sinusoidal equation to the observed fat oxidation rates and takes into account the dilation, symmetry and translation of the fitted curve (Chenevière et al. 2009). This model estimate was achieved through an excel spreadsheet that involved a solver function kindly provided by Dr Xavier Chenevière].

### Metabolic measurements

Expired gas samples were collected into 100–150 L Douglas bags (Cranlea and Hans Rudolph, Birmingham, UK) via a mouthpiece connected to a two-way, T-shaped non-rebreathing valve (Model 2700, Hans Rudolph Inc, Kansas City, USA) and Falconia tubing (Hans Rudolph Inc, Kansas City, USA). Concentrations of O_2_ and CO_2_ were measured in a known volume of each sample via paramagnetic and infrared transducers, respectively (Mini MP 5200, Servomex Group Ltd., Crowborough, East Sussex, UK) and until values were stable. The sensors were calibrated to a two-point low and high calibration of known gas concentrations (low: 99.998% nitrogen, 0% O_2_ and CO_2_; high: balance nitrogen mix, 20.06% O_2_, 8.11% CO_2_) (BOC Industrial Gases, Linde AG, Munich, Germany). Concurrent measurements of inspired air composition were made during collections of expired gas samples to adjust for changes in ambient O_2_ and CO_2_ concentrations (Betts and Thompson, 2012). Indirect calorimetry was used to determine: $\dot{V}$O_2_ (L·min^−1^); $\dot{V}$CO_2_ (L·min^−1^); and rate of fat oxidation [g·min^−1^; estimated by Frayn’s stoichiometric equations assuming urinary nitrogen excretion was negligible (Frayn, 1983)].

Resting metabolic rate [(RMR; kcal·day^−1^) and resting rates of fat oxidation (g·min^−1^)] were measured following guidelines for best practice (Compher et al. 2006): after 15 min of quiet rest in a semi-supine position, RMR was measured by indirect calorimetry of at least two expired gas samples of five-min duration and within 100 kcal·day^−1^.

### Habitual lifestyle assessment

Habitual physical activity levels were assessed by asking participants to wear a physical activity monitor (Actiheart™, Cambridge Neurotechnology, Papworth, UK) over the7 days prior to Trial A. Ideally, a minimum of four valid days (monitor worn for ≥ 90% of time in a day and < 30% of no heart rate signal) was required to determine habitual physical activity levels (excluding *n* = 5 participants for whom only three valid days were available). Additionally, energy expenditure and heart rate values from rest and the FAT_MAX_ test were entered in the Actiheart™ software to derive an individually calibrated model estimate of physical activity energy expenditure (kcal·day^−1^) and mins per day spent in different physical activity thresholds. To assess pre-trial physical activity standardisation, the monitor was also worn for the 48 h before Trial A and Trial B. Habitual energy and macronutrient intake were assessed by a self-weighed diet diary. Participants were provided with a set of scales (Pro Pocket Scale_TOP2KG_, Smart Weigh Scales) and asked to keep a written record of their food and fluid intake for at least 4 days in the week preceding Trial A (including at least one weekend day). Additionally, the two days immediately prior to Trial A were recorded, so that participants could replicate this on the two days prior to Trial B. Diet records were analysed using Nutritics software (Nutritics Ltd., Dublin, Ireland).

### Statistical analysis

Assumptions (normality, heteroscedasticity, linearity and proportional bias) for the below statistical tests were explored by a combination of visual inspection (histograms, skewness and kurtosis values and scatter graphs) and quantitative statistical tests (Shapiro–Wilk test, correlations, Levene’s test, Mauchly's Test of Sphericity) on raw data and residuals of comparisons. Parametric statistical tests were conducted when assumptions were met with either transformation (natural logarithm followed by anti (inverse)-log to facilitate the interpretation of data in their raw units), or the appropriate non-parametric equivalent was performed. ANOVA models were conducted irrespective of normality due to robustness against violations of normality (Maxwell 1990).

A range of a priori statistical analysis tests were performed to assess the day-to-day reliability of PFO (g·min^−1^) and FAT_MAX_ (%$\dot{V}$O_2_peak) as advocated (Atkinson and Nevill 1998): (1) systematic bias was assessed by dependent sample *t* tests and mixed-design analysis of variance (within-subject: Trial A and Trial B; between-subject: group category as per below). Bonferroni-adjusted *p* values were applied to control for multiple comparisons and for when significant main or interaction effects were detected in the ANOVA models; (2) an index of relative reliability was obtained by bivariate correlation (Pearson correlation coefficient; *r*); (3) the absolute day-to-day reliability was investigated by within-subject coefficient of variation [CV; root mean square method(Bland 2006)]; typical error [TE; SD of difference between scores/√2 (Hopkins 2015a)]; and Bland–Altman plot with mean difference (bias) and 95% limits of agreement (LoA) (Bland and Altman 1986). Mean difference was calculated by Trial A minus Trial B; and (4) individual data were plotted on graphs (as shown in Supplementary figures).

These tests were performed on the whole sample and on a range of sub-group analyses:

i. Whole sample (*n* = 97). Systematic bias was assessed by dependent sample *t* tests. As PFO and FAT_MAX_ were not available for *n* = 2 participants in one or both trials (participant fainting and hyperventilation, respectively), these participants were excluded, leaving a maximum sample size of *n* = 97.
ii. Data analysis approach (MV, P2 and SIN; *n* = 72; *n* = 34 females). A two-way repeated measures ANOVA (within subject; Trial: Trial A and Trial B; Model: MV, P2 and SIN) was performed for this analysis. This analysis primarily investigated the day-to-day reliability of each individual data analysis approach rather than the level of agreement between modelling approaches. Mathematical modelling could not be performed for *n* = 25 participants due to lack of fat oxidation data points or a plateau in data.
iii. Sex (*n* = 50 males and 47 females). Participants were divided into male and female based on self-report from a participant questionnaire.
iv. Cardiorespiratory fitness (*n* = 97). Participants were categorised into three training classifications (untrained, recreationally trained, highly trained) based on the corresponding $\dot{V}$O_2_peak thresholds outlined for males and females (De Pauw et al. 2013; Decroix et al. 2016). Due to the low sample size (*n* = 2), the highly trained group was excluded from reliability statistics.
v. Fat Mass Index (*n* = 96). Participants were classified into four categories (fat deficient, *healthy*, excess adiposity and obese) as identified by Kelly et al. (2009). Due to only one participant being classified as obese, this individual was excluded from this respective sub-group analysis.
vi. Physical activity level (*n* = 94). Participants were categorised into four physical activity level classifications (sedentary, low active, moderately active, very active) as identified by Brooks et al. (2004). Physical activity data were not available for *n* = 3 participants and due to the low sample size (*n* = 3), the sedentary group was excluded from reliability statistics.
vii. Menstrual cycle status and contraceptive use (females only, *n* = 47). Female participants were divided into seven categories [menstrual cycle matched (Trial A and Trial B occurred in the same phase of the menstrual cycle verified by progesterone concentrations), menstrual cycle non-matched (Trial A and Trial B occurred in different phases of the menstrual cycle phase verified by progesterone concentrations), unknown (eumenorrheic but stage of the menstrual cycle when Trial A and Trial B took place was unknown), contraceptive use combined pill, contraceptive use progesterone-only pill, contraceptive use intrauterine device (IUD), contraceptive use intrauterine system (IUS) and post-menopausal]. Due to the low sample sizes in the progesterone-only pill, IUD, IUS and post-menopausal categories (*n* = 4, 5, 3 and 3, respectively), these sub-groups were excluded from reliability analyses.

Additionally, the above statistical tests were also employed to explore the level of agreement between the three analysis approaches (MV, P2 and SIN) to determine PFO and FAT_MAX_. Estimates of PFO and FAT_MAX_ represent the average of Trial A and Trial B, where a one-way ANOVA [within-subject (three levels): MV, P2 and SIN] was used to assess model differences and systematic bias. The sample size for this analysis was *n* = 72 (*n* = 34 females).

Log transformation and antilog were required for FAT_MAX_ analyses of: (1) whole sample, (2) data analysis approach (reliability of individual models), (3) sex, (4) cardiorespiratory fitness ($\dot{V}$O_2_peak), and (5) physical activity level. Readers should note that the interpretation of these analyses is distinctly different from when log-transformation was not performed (see Supplementary material 1A for a description). Pearson correlation coefficient, TE and CV were computed for logged data via analysis recommended by Hopkins (2015b). When transformation did not improve the proportional bias (differences plotted against mean) and/or heteroscedasticity (absolute differences plotted against mean) in the data (or consistently across sub-groups), the raw non-transformed data were used for analysis (as such, more caution is required for the interpretation of these results). This was apparent for FAT_MAX_ analysis of: (1) fat mass index, (2) menstrual cycle status and contraceptive use, and (3) level of agreement between data analysis approaches. Pearson correlation coefficients were interpreted by an *r* of < 0.40, 0.40–0.74 and ≥ 0.75 for poor, fair to high and excellent, respectively (Dandanell et al. 2017a, b). There is no consensus to date on what constitutes an acceptable level of reproducibility for CVs, TEs or 95% LoAs for PFO and FAT_MAX_. However a mean CV of 8% and 11% for the day-to-day reliability of PFO and FAT_MAX_ have been previously stated as acceptable (Hansen et al. 2019). Additionally, Nordby et al. (2015) and Rosenkilde et al. (2015) report an exercise training-induced increase in PFO and FAT_MAX_ of ~ 0.13 to 0.16 g·min^−1^ and 5–8%$\dot{V}$O_2_peak, respectively, compared to non-exercising control groups. Thus, these values were used to help interpret the day-to-day variability values produced for CVs and particularly 95% LoAs in PFO and FAT_MAX_.

Additionally, prior to any of the above analyses, a sensitivity analysis performed in women found that the differences in concentrations of oestradiol and progesterone between Trial A and Trial B did not affect estimates of PFO and FAT_MAX_ (see Supplementary material 1B). This was performed due to the speculation that substrate utilisation during exercise may differ across the menstrual cycle only if concentrations of oestrogen differ by twofold or more between testing occasions (Oosthuyse and Bosch 2010). Consequently, a sensitivity analysis also found no differences in the interpretation of results from menstrual cycle status and contraceptive use when the above statistical tests were performed with and without individuals whose concentrations of oestradiol and progesterone were ≥ two- and < twofold between trials, respectively.

Descriptive and statistical analyses were run on Microsoft Excel (2013) and IBM SPSS statistics version 25 for windows (IBM, New York, USA) and graphs were created on Graph Pad Prism 7 software (La Jolla, CA, USA). Data are presented as means ± SD (or 95% confidence intervals for *r*, CV and TE) unless otherwise stated and statistical significance was accepted at *p* ≤ 0.05.

## Results

### Day-to-day reliability of PFO and FAT_MAX_

#### Whole sample

**Fig. 1:**
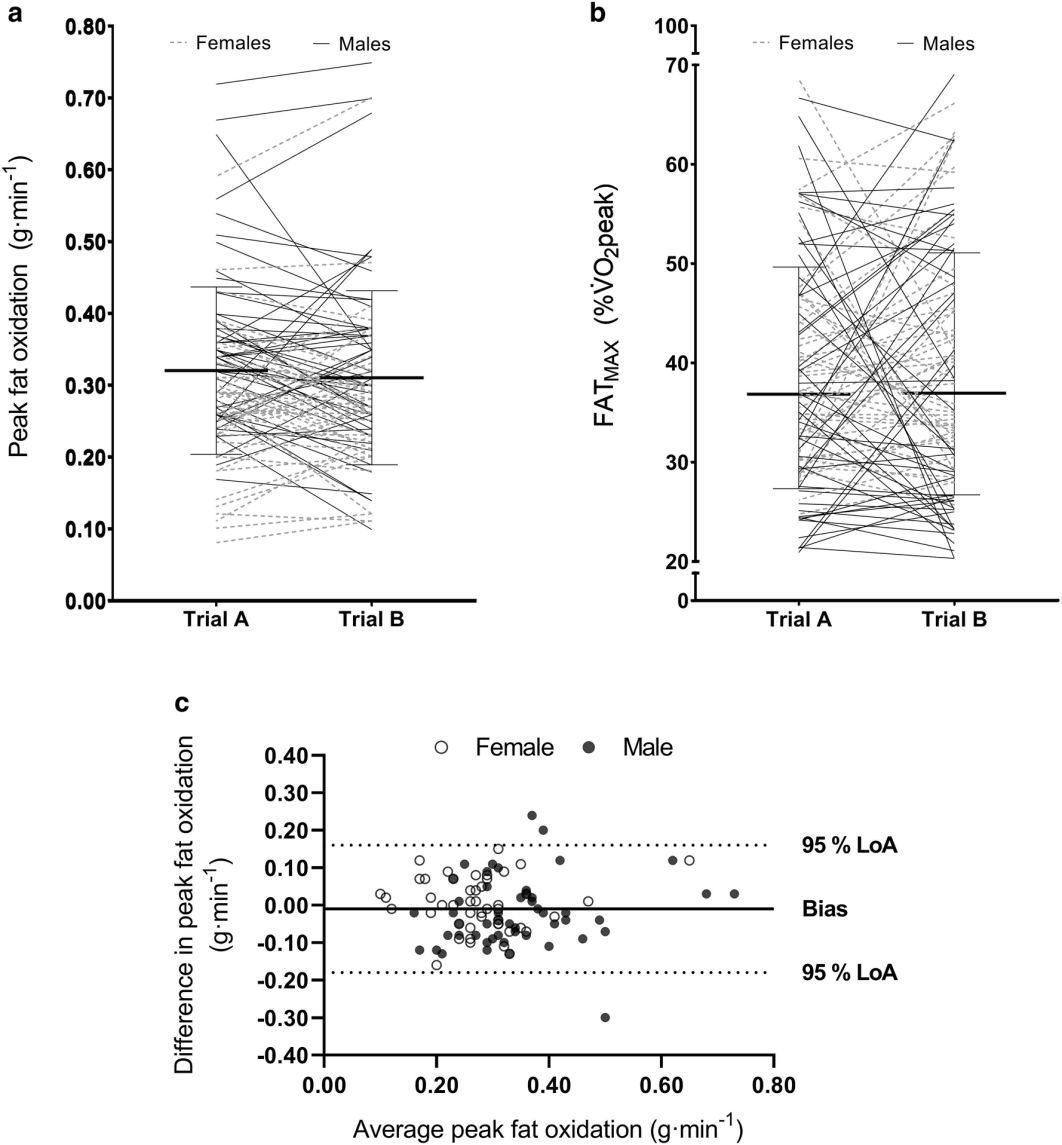
Comparison of Trial A and Trial B for peak fat oxidation rate (**a**; g·min^−1^) and FAT_MAX_ (**b** %$\dot{V}$O_2_peak) in all participants (whole sample). The solid thick line represents mean ± SD (or × / ÷ for FAT_MAX_) with individual data denoted by the thin lines (dashed = Females; solid = Males). **c** A Bland–Altman plot displaying the difference in PFO (g·min^−1^) between Trial A and B. The solid line represents bias and the dashed lines represent lower and upper 95% limits of agreement. Females are denoted by open circles and males are indicated by filled circles. Measured values approach used to determine PFO and FAT_MAX_

No systematic bias was evident between Trial A and Trial B for PFO (Fig. 1a; *p* = 0.791) or FAT_MAX_ (*p* = 0.919; Fig. 1b). The absolute reliability (TE, CV and 95% LoAs) of both measures was low (Fig. 1c; Table 3) with high and fair relative reliability (*r*) for PFO and FAT_MAX_, respectively (Table 3).

**Table 3:** Whole sample day-to-day reliability in peak fat oxidation and FAT_MAX_

	A	B	Avg
Peak fat oxidation (g·min^−1^; *n* = 97)			
Mean ± SD	0.32 ± 0.12	0.31 ± 0.12	0.31 ± 0.11
Bias ± SD (g·min^−1^)	− 0.01 ± 0.08		
*r*	0.75 (0.65–0.82)		
CV (%)	21 (17–24)		
TE (g·min^−1^)	0.06 (0.05–0.07)		
95% LoA (± ; g·min^−1^)	0.17		
FAT_MAX_ (*n* = 97)			
Mean × / ÷ SD	37 × / ÷ 1.35	37 × / ÷ 1.38	37 × / ÷ 1.30
Bias ratio	1.00		
*r*	0.45 (0.30–0.57)		
CV (%)	26.0 (23.0–30.0)		
TE ratio	1.26 (1.23–1.30)		
95% ratio LoA (× / ÷)	1.90		

Peak fat oxidation data presented as mean (± 95% CI) unless otherwise stated; A = Trial A; B = Trial B; FAT_MAX_ data are transformed and presented as mean (× / ÷ 95% CI) unless otherwise stated

*SD* standard deviation, *Avg* Average of Trial A and B (mean ± SD) ratio, *r* Pearson correlation, *CV* within-subject coefficient of variation, *TE* typical error, *LoA* limits of agreement, *FAT*_*MAX*_* LoA* SD ratios

#### Data analysis approach

A significant main effect of data analysis approach (MV, P2 and SIN) was found for PFO (*p* < 0.001) and FAT_MAX_ (*p* = 0.001). Post hoc tests revealed P2 produced significantly lower and higher estimates of PFO and FAT_MAX_, respectively, at the group level compared to MV (PFO, *p* < 0.001; FAT_MAX_* p* = 0.026) and SIN (both *p*’s ≤ 0.001) but there were no differences between MV and SIN (PFO, *p* = 0.653; FAT_MAX_, *p* = 1.000) (Supplementary Table 2). No main effects of trial (*p* = 0.576 and 0.768) nor trial*data analysis approach interaction effects (*p* = 0.737 and 0.767) were apparent for PFO and FAT_MAX_, respectively. No systematic bias was evident for PFO (*p* values > 0.482) nor for FAT_MAX_ (*p* values > 0.329).

There was large absolute day-to-day variability among all the data analysis approaches for PFO whilst the relative reliability was high (Supplementary Table 2; Supplementary Fig. 2a, 2b and 2c). The absolute day-to-day variability was large for FAT_MAX_ across all the data analysis approaches with the MV approach displaying the greatest variation alongside the relative reliability of approaches ranging from fair to high (Supplementary Table2; and Supplementary Fig. 3a, 3b and 3c).

#### Sex

A significant main effect of sex was detected for PFO when expressed in absolute terms (g·min^−1^*; p* < 0.001) but not for FAT_MAX_ (*p* = 0.070) indicating that men had a higher absolute PFO than women (*p* < 0.001). Otherwise, no main effects of trial (*p* = 0.268 and 0.931) nor trial*sex interaction effects (*p* = 0.169; and 0.353) were found for PFO (g·min^−1^) and FAT_MAX_. No systematic bias was detected in either men (*p* = 0.380 and *p* = 0.603) or women (*p* = 0.743 and *p* = 0.373) for PFO (g·min^−1^) and FAT_MAX,_ respectively (Supplementary Table 3).

The absolute day-to-day reliability in PFO (g·min^−1^) was poor for both men and women with high relative reliability evident (Supplementary Table 3; Supplementary Fig. 2d). Low day-to-day reliability in FAT_MAX_ was apparent for both sexes where males displayed slightly greater absolute variation compared to females (Supplementary Table 3; Supplementary Fig. 3d). The relative reliability of FAT_MAX_ was fair in both men and women (Supplementary Table 3).

#### Cardiorespiratory fitness

A significant main effect of group was found for PFO (*p* < 0.001) but not for FAT_MAX_ (*p* = 0.098) showing that trained individuals had a higher PFO compared to untrained individuals (Supplementary Table 4). There was no main effect of trial (*p* = 0.182 and 0.866) nor trial*group interaction effects (*p* = 0.836 and 0.229) for PFO and FAT_MAX_, respectively. No systematic bias was found in untrained (*p* = 0.297 and 0.318) or trained individuals (*p* = 0.395 and 0.459) for PFO and FAT_MAX,_ respectively.

There was low absolute day-to-day reliability for PFO in both untrained and trained individuals in addition to high relative reliability (Supplementary Table 4; Supplementary Fig. 2e). For FAT_MAX_, absolute variation was high and was greater in trained *versus* untrained individuals (Supplementary Table 4; Supplementary Fig. 3e) with poor and fair relative reliability evident, respectively.

*Fat mass index* A significant main effect of group was detected for PFO and FAT_MAX_ (*p* = 0.001 and 0.013, respectively) indicating that individuals who were fat deficient had a higher PFO and FAT_MAX_ than individuals classified with *healthy* levels of adiposity (*p* = 0.001) (Supplementary Table 5). There were no main effects of trial (*p* = 0.418 and 0.561) nor trial*group interaction effects (*p* = 0.526 and 0.268) for PFO or FAT_MAX_, respectively. There was no evidence of systematic bias across the groups for either PFO (*p* values > 0.112) or FAT_MAX_ (*p* values > 0.221).

The absolute reliability for PFO showed a slight step-like fashion, whereby individuals classified as fat deficient displayed the highest absolute variability and individuals with excess adiposity showed the lowest, albeit large overlapping of the 95% CI are evident (Supplementary Table 5; Supplementary Fig. 2f). Alternatively, high to excellent relative reliability was apparent across the FMI classifications (range of *r* = 0.66–0.81; Supplementary Table 5). This step-like fashion in estimates of absolute reliability was less apparent for FAT_MAX_ with similarly high variation for individuals with *healthy* and excess adiposity levels which was slightly greater in individuals categorised as fat deficient (Supplementary Table 5; Supplementary Fig. 3f). The relative reliability of FAT_MAX_ ranged from poor to fair (range of *r* = 0.19–0.49).

#### Physical activity level

A significant main effect of group was apparent for PFO (*p* = 0.003) but not FAT_MAX_ (*p* = 0.130) with post hoc tests revealing that individuals with low habitual physical activity levels had a lower PFO than very active individuals (*p* = 0.002; Supplementary Table 6). No main effects of trial (*p* = 0.094 and 0.776) nor trial*group interaction effects (*p* = 0.929 and 0.205) were found for PFO and FAT_MAX_, respectively. No systematic bias was evident across either of the groups for either PFO (*p* values > 0.142) or FAT_MAX_ (all Bonferroni-adjusted *p* values > 0.016).

Low absolute reliability of PFO was similarly evident across active individuals and those with low levels of habitual physical activity with a slightly higher TE and 95% LoA apparent in very active individuals (Supplementary Table 6; Supplementary Fig. 2g). The relative reliability for PFO was high across all levels of habitual physical activity level (range 0.73–0.74). Alternatively, greater absolute day-to-day variability for FAT_MAX_ was apparent in active and very active individuals compared to individuals with low levels of habitual physical activity (Supplementary Table 6; Supplementary Fig. 3 g). Fair relative reliability was evident for FAT_MAX_ across all habitual physical activity levels (range 0.43–0.57).

#### Menstrual cycle status and contraceptive use

No significant main effects of trial (*p* = 0.636 and 0.495), group (*p* = 0.385 and 0.279) nor trial*group interaction effects (*p* = 0.762 and 0.184) were apparent for PFO and FAT_MAX_, respectively (Supplementary Table 7). There was no systematic bias across any of the groups for either PFO (*p* values > 0.299) or FAT_MAX_ (*p* values > 0.090).

Similarly low absolute day-to-day reliability was apparent across all groups for PFO aside for women whose menstrual cycle phase was matched between Trial A and B who displayed a greater CV and 95% LoA (Supplementary Table 7; Supplementary Fig. 2h). The relative reliability of PFO across all groups ranged from fair to excellent (Supplementary Table 7). The absolute variability between Trial A and B for FAT_MAX_ was similar between women whose menstrual cycle phase was matched or not known, but women who used the combined pill for contraception or whose menstrual cycle phase was not matched between trials displayed lower absolute reliability to a similar magnitude for FAT_MAX_ (Supplementary Table 7; Supplementary Fig. 3h). Moreover, excellent relative reliability for FAT_MAX_ was apparent for women whose menstrual cycle phase was matched and for women who were unmatched (or not known) between Trial A and B, with fair and poor relative reliability found for women whose menstrual cycle was not matched or used the combined pill for contraception, respectively (Supplementary Table 7).

## Agreement between data analysis approaches

As identified above, significant main effects of the data analysis approach applied to determine PFO (*p* < 0.001) and FAT_MAX_ (*p* = 0.006) were found (Table 4). As FAT_MAX_ data were not log-transformed for agreement between data analysis approaches, post hoc tests indicated that P2 produced slightly higher estimates of FAT_MAX_ compared to SIN (*p* < 0.001) but not MV (*p* = 0.692). No systematic differences were found between MV and SIN for FAT_MAX_ (*p* = 0.125). This was confirmed by dependent sample *t* tests that found P2 had modestly lower PFO estimates compared to MV and SIN (both *p*’s < 0.001) and a slightly greater FAT_MAX_ estimate compared to SIN (*p* < 0.001). Additionally, FAT_MAX_ was modestly higher with MV versus SIN (*p* = 0.042).

**Table 4:** Level of agreement between data analysis approaches to determine peak fat oxidation and FAT_MAX_

	MV-P2			MV-SIN			P2-SIN		
	MV	P2	Avg	MV	SIN	Avg	P2	SIN	Avg
Peak fat oxidation (g·min^−1^; *n* = 72)									
Mean ± SD	0.32 ± 0.10	0.30 ± 0.10^***^	0.31 ± 0.10	0.32 ± 0.10	0.31 ± 0.10	0.32 ± 0.10	0.30 ± 0.10^***^	0.31 ± 0.10	0.31 ± 0.10
Bias ± SD (g·min^−1^)	− 0.02 ± 0.02^§*^			0.00 ± 0.03			0.01 ± 0.02^§*^		
*R*	0.97 (0.95–0.98)			0.96 (0.93–0.97)			0.97 (0.96–0.98)		
CV (%)	7 (5–8)			6 (3–8)			5 (0–8)		
TE (g·min^−1^)	0.02 (0.01–0.02)			0.02 (0.02–0.02)			0.02 (0.01–0.02)		
95% LoA (± ; g·min^−1^)	0.05			0.06			0.05		
FAT_MAX_ (*n* = 72)									
Mean ± SD	39 ± 11	40 ± 7	39 ± 8	39 ± 11	37 ± 8	38 ± 8	40 ± 7	37 ± 8	39 ± 8^||***^
Bias ± SD (%$\dot{V}$O_2_peak)	1 ± 7			− 1 ± 5			− 2 ± 4		
*r*	0.79 (0.68–0.86)			0.87 (0.80–0.92)			0.90 (0.84–0.93)		
CV (%)	13 (10–15)			10 (8–12)			9 (7–10)		
TE (g·min^−1^)	5 (4–6)			4 (3–4)			3 (2–3)		
95% LoA (± %$\dot{V}$O_2_peak)	13			10			7		

Data presented as mean (± 95% CI) unless otherwise stated; *n* = 34 and 38 females and males, respectively

*A* Trial A, *B* Trial B, *SD* standard deviation, *Avg* Average of Trial A and B (mean ± SD), *r* Pearson correlation, CV within-subject coefficient of variation, *TE* typical error, *LoA* limits of agreement

***p < .001, P2 vs MV and SIN; ^§*^p < 0.001; ||***p < .001, P2 vs SIN

**Fig. 2:**
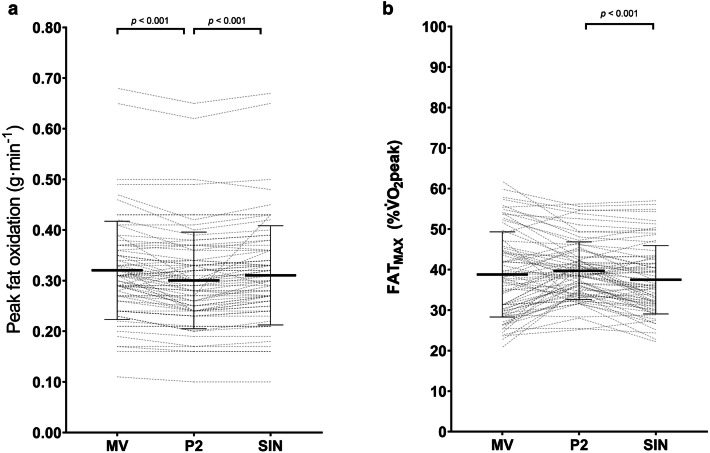
Comparison of peak fat oxidation (g·min^−1^; **a**) and FAT_MAX_ (%$\dot{V}$O_2_peak; **b**) between the different data analysis approaches applied to determine PFO and FAT_MAX_ (values reflect an average of Trial A and Trial B). The solid thick line represents mean ± SD with individual data denoted by the thin lines

The absolute agreement between the data analysis approaches to determine PFO was high (as indicated by the low values of CVs, TEs and 95% LoAs) with excellent relative reliability also evident (Table 4; Fig. 2). The absolute agreement in FAT_MAX_ was similarly high between data analysis approaches, albeit comparisons involving the MV approach were modestly lower (Table 4; Fig. 2). The relative agreement between all three approaches was excellent FAT_MAX_ (Table 4).

## Discussion

The main objective of this study was to explore the day-to-day reliability of PFO and FAT_MAX_ in a diverse sample of healthy men and women. The overall findings were that PFO and FAT_MAX_ display poor day-to-day reliability in a heterogeneous population of healthy adults, as evident by the reported typical errors (± 0.06 g·min^−1^ and × / ÷ 1.26%$\dot{V}$O_2_peak, respectively), CVs (> 20%) and large 95% LoA (± 0.17 g·min^−1^ and × / ÷ 1.90%$\dot{V}$O_2_peak, respectively). This large day-to-day variability was apparent despite no evidence of systematic bias for PFO and FAT_MAX_ (− 0.01 g·min^−1^ and 0%$\dot{V}$O_2_peak, respectively). Moreover, these findings are predominantly independent of sex, cardiorespiratory fitness, fat mass index, physical activity level and menstrual cycle status (and contraceptive use) as similar levels of variability in PFO and FAT_MAX_ were reported across these sub-groups. Additionally, while similar levels of agreement were apparent between the data analysis methods to estimate PFO and FAT_MAX_, larger day-to-day variability—particularly in FAT_MAX_—was apparent when the MV data analysis approach was applied.

The day-to-day reliability of PFO and FAT_MAX_ observed in this study is similar to that reported by some (Croci et al. 2014; Dandanell et al. 2017a, b; Meyer et al. 2009) but not all prior studies (De Souza Silveira et al. 2016; Hansen et al. 2019; Marzouki et al. 2014). For example, Croci et al. (2014) reported large 95% LoAs (range ± 0.24–0.26 g·min^−1^ and 27–32%$\dot{V}$O_2_peak) and CVs (> 15%) for both PFO and FAT_MAX_ during cycle ergometry across three different data analysis approaches (MV, P3 and SIN) in fifteen recreationally trained males. The present study extends the generalisability of these findings to a large diverse sample of healthy men and women by reporting similar day-to-day variability, particularly for PFO, across the whole sample (MV approach only) and the three data analysis approaches used to determine PFO and FAT_MAX_ (Supplementary Fig. 2a–c, 3a–c; Supplementary Table 2, respectively). The larger day-to-day variability reported here and by Croci et al. (2014) compared to some previous studies may be due to differences in methodology (e.g. FAT_MAX_ protocol, gas analysis equipment, data analysis techniques and pre-trial standardisation). Indeed, this study assessed the day-to-day reliability of PFO and FAT_MAX_ by use of the Douglas bag technique and a Servomex gas analyser which may display different day-to-day and/or measurement-to-measurement reliability of gas exchange data compared to breath-by-breath gas analysis systems. Accordingly, there is a need for direct comparisons of populations and methods within-studies in order to establish whether these factors predominantly explain the discrepancies between studies.

The present study does suggest though that any differences in the populations recruited by prior PFO reliability studies are not likely significant contributing factors to the differences reported in the day-to-day reliability of PFO and FAT_MAX_. The relatively large sample size recruited in the present study (maximum *n* = 97 for analyses) facilitated various sub-group analyses, allowing direct comparisons of data collected by the same methods. Whilst better day-to-day reliability was apparent in some sub-groups for both PFO and FAT_MAX_, all sub-groups (excluding females whose menstrual cycle phase was unknown) had quite large 95% LoAs [> ± 0.10 g·min^−1^ and 10%$\dot{V}$O_2_peak (or × / ÷ 1.46)], TEs [0.04 g·min^−1^ and 8%$\dot{V}$O_2_peak (or × / ÷ 1.15)] and CVs (> 13%) for PFO and FAT_MAX_, respectively. Furthermore, the present study found that controlling for the menstrual cycle phase (objectively verified) and/or contraceptive use through the combined pill had no clear impact on group mean estimates nor the day-to-day reliability of PFO and FAT_MAX_ (Supplementary Table 7; Supplementary Figs. 2h, 3h, respectively). From a practical perspective, this suggests that controlling for menstrual cycle phase may not be an important requirement in studies assessing PFO, which is in agreement with recent findings by Frandsen et al. (2020). Thus, more future studies can recruit female participants without using this as justification for their exclusion. This noted, whilst oestradiol is the main circulating form of oestrogen (Mauvais-Jarvis et al. 2013), we did not assess total oestrogen concentrations per se. Furthermore, the ratio of oestrogen-to-progesterone may also impact substrate use during exercise (Oosthuyse and Bosch 2010) but was not explored here. Whilst the absence of any systematic bias (e.g. learning effects) in estimates of PFO and FAT_MAX_ also suggests there may be no need to perform a familiarisation session prior to the assessment of peak fat oxidation, repeated assessment is still required given the large day-to-day variation in PFO reported here.

Some caution should be applied in the interpretation of the reproducibility of FAT_MAX_ in sub-group analyses. This is because the MV approach, which was adopted to facilitate larger sample sizes for the sub-group analyses, showed lower day-to-day reliability in FAT_MAX_ compared to P2 and SIN (Supplementary Table 2; Supplementary Fig. 3a). This greater variability apparent in FAT_MAX_ with MV, may arise from the fact that the MV approach can be highly influenced when two or more recorded fat oxidation rates at different exercise intensities provide similar values. In contrast, mathematical models (e.g. P2, P3 and SIN) are largely immune to this issue and thus, are better suited to analysing data that does not form a clear parabolic curve with a distinct peak in fat oxidation rates (Chenevière et al. 2009). Thus, due to their better reproducibility, mathematical models are recommended when the assessment of FAT_MAX_ is a key focus.

The current study further adds to the literature by reporting similar levels of agreement between the three data analysis approaches that were applied to determine PFO and FAT_MAX_ (Table 4). These findings are largely consistent with the only prior study to have also applied the full range of agreement statistics available to investigate this (Chenevière et al. 2009). However, Chenevière et al. (2009) did find higher levels of agreement between P3 and SIN compared to the present study, reporting a mean bias of zero (~ 0.00 g·min^−1^ and ~ 0%$\dot{V}$O_2_peak) and extremely narrow limits of agreement (~ 0.01 g·min^−1^ ~ 2%$\dot{V}$O_2_peak) for PFO and FAT_MAX_, respectively. Additionally, the present study found that P2 and SIN modestly, but systematically underestimated group mean estimates of PFO and FAT_MAX_, respectively, when data analysis approaches were compared (Table 4). Interestingly, the direction and magnitude of differences in PFO and FAT_MAX_ between approaches were similar to those reported by Chenevière et al. (2009), suggesting that these slight discrepancies may in part be accounted for by the present study being sufficiently powered to statistically detect these differences. Importantly, however, discrepancies do not appear to be an artefact of the polynomial order selected (i.e. P2 versus P3) as no systematic differences in estimates of PFO or FAT_MAX_ between P2 and P3 have been detected (Dandanell et al. 2017a, b). Nonetheless, given the array of data analysis approaches applied in the literature (Amaro-Gahete et al. 2019), the evidence to date collectively suggests that relatively similar estimates of PFO and FAT_MAX_ are obtained independent of the data analysis approach applied. Moreover, similar reproducibility particularly when determining PFO appears to be apparent among the most widely used and recommended data analysis approaches (i.e. MV, SIN and polynomial modelling).

The high day-to-day variability in PFO and FAT_MAX_ reported here and previously (Croci et al. 2014; Dandanell et al. 2017a, b) may partly be accounted for by differences in pre-trial standardisation procedures (Astorino and Schubert 2017). Indeed, fuel selection kinetics during exercise are influenced by many factors, such as immediate nutrient status (Gonzalez et al. 2013), habitual dietary macronutrient composition (Støa et al. 2016) and chronic/acute physical activity levels (Venables et al. 2005). As per the recommendations (Astorino and Schubert 2017), participants were asked to replicate their dietary intake and physical activity levels, alongside avoiding vigorous physical activity, over the 48 h prior to each test. The lack of a strict controlled diet in the 48 h prior to testing in this study may have added to the day-to-day variability of PFO, potentially by altering pre-exercise muscle glycogen levels (Maunder et al. 2018). Equally, whilst this study attempted to objectively verify physical activity standardisation via the wearing of a physical activity monitor, due to data quality issues (e.g. monitor not worn or insufficient data traces), objective verification was not possible for many participants (*n* = 63). In subjects for whom data were available (*n* = 36), no participant replicated their total physical activity energy expenditure (kcal·day^−1^) or estimated time spent in activity intensity thresholds when an arbitrary threshold of ± 10% of Trial A was set. In addition, only seven participants avoided vigorous physical activity during this period. This demonstrates not only the difficulty of capturing physical activity levels across a short timeframe, but also that self-report confirmation is not sufficient to ensure pre-trial physical activity standardisation (i.e. objective assessment is necessary), which likely contributes to the day-to-day variability in PFO and FAT_MAX_.

## Conclusion

The present study demonstrates that large day-to-day variability is present when estimating PFO and FAT_MAX_ in a heterogeneous cohort of healthy men and women. Moreover, this low reproducibility is consistent across sex and different levels of cardiorespiratory fitness, fat mass indices, physical activity levels, and menstrual cycle status and contraceptive use through the combined pill. Nevertheless, there is little-to-no evidence of systematic bias in measures of peak fat oxidation across two identical testing sessions, suggesting there is no need to conduct a familiarisation session. Additionally, the data analysis approach used to estimate PFO and FAT_MAX_ does not appear to affect reliability estimates particularly for PFO, with similar levels of agreement apparent between the MV, P2 and SIN approaches. Collectively, this suggests that future studies should perform repeated assessments to more accurately determine PFO and FAT_MAX_. This will help more precisely prescribe exercise training upon and/or explore the practical relevance of PFO and FAT_MAX_ for health and/or endurance exercise performance.

## Electronic supplementary material

Below is the link to the electronic supplementary material.

Supplementary file1 (DOCX 42 kb)

Supplementary file2 (JPG 833 kb)

Supplementary file3 (JPG 797 kb)

## Abbreviations

CVs: Coefficients of variation
DEXA: Dual-energy X-ray absorptiometry
ELISA: Enzyme-linked immunosorbent assay
FAT_MAX_: The intensity that elicits PFO
IUD: Intraauterine device
IUS: Intraauterine system
LoAs: Limit of agreements
MV: Measured values
NEFA: Non-esterified fatty acids
P2: Second-order polynomial curve
P3: Third-order polynomial curve
PFO: Peak fat oxidation
*r*: Pearson correlation coefficient
RMR: Resting metabolic rate
SD: Standard deviation
SIN: SINE model
TE: Typical error
$$\dot{V}$$˙O_2_: Oxygen consumption
