# Supplementary material for: The day-to-day reliability of peak fat oxidation and FATMAX
Source: Eur J Appl Physiol. 2020 Jun 1;120(8):1745–59. doi: 10.1007/s00421-020-04397-3 (PMC7340634; doi:10.1007/s00421-020-04397-3)
Supplement: Supplementary file 1 — Supplementary file1 (DOCX 42 kb) [file 421_2020_4397_MOESM1_ESM.docx]

**TITLE:** The day-to-day reliability of peak fat oxidation and FAT_MAX_

**AUTHORS:** Oliver J Chrzanowski-Smith^1^, Robert M Edinburgh^1^, Mark P Thomas^1^, Nicos Haralabidis^1^, Sean Williams^1^, James A Betts^1^, Javier T Gonzalez^1^

**CORRESPONDENCE:** Dr Javier T. Gonzalez

Department for Health

University of Bath

Bath, BA2 7AY

United Kingdom

Tel: +44-1225-385-518

Email: J.T.Gonzalez@bath.ac.uk

**AFFILIATIONS:** ^1^ Department for Health, University of Bath, Bath, BA2 7AY, UK

O.J.Chrzanowski-Smith@bath.ac.uk

R.M.Edinburgh@bath.ac.uk

M.Thomas@bath.ac.uk

nh546@bath.ac.uk

S.Williams@bath.ac.uk

J.Betts@bath.ac.uk

J.T.Gonzalez@bath.ac.uk

**RUNNING HEAD:** Reliability of peak fat oxidation and FAT_MAX_

**Supplementary material 1**

**Supplementary material 1A. Interpretation of log transformed and anti-log data for typical error ratio, mean ratio bias and 95% ratio limits of agreement.**

A typical error ratio of 1.90 is interpretable as, on average, Trial B FAT_MAX_ estimates were ×/÷ 1.90 higher or lower, respectively compared to Trial A FAT_MAX_ estimates.

A mean ratio bias of 0.92 is interpretable as, on average, Trial B estimates of FAT_MAX_ were 92% of Trial A FAT_MAX_ estimates. A 95% ratio limit of agreement SD of ×/÷ 1.46 implies that ~ 95% of Trial B FAT_MAX_ estimates were up to 42% higher and 34% lower than Trial A FAT_MAX_ estimates, assuming a mean ratio bias of 0.92.

**Supplementary material 1B. Oestradiol and Progesterone concentration sensitivity analysis**

For this analysis female participants, who had data on concentrations of oestradiol and progesterone on Trial A and Trial B were categorised into one of four categories based on the difference in concentrations of these hormones between Trial A and B:

1. Oestradiol and progesterone concentrations ≥ and < two-fold, respectively (*n* = 5).
2. Oestradiol and progesterone concentrations both < two-fold (*n* = 14)
3. Oestradiol and progesterone concentrations both ≥ two-fold (*n* = 9)
4. Oestradiol and progesterone concentrations < and ≥ two-fold, respectively (*n* = 4)

This grouping was completed solely on concentrations of oestradiol and progesterone and thus, was independent of reproductive status and contraceptive use. This analysis was conducted due to speculation in the literature that if oestrogen concentrations are ≥ two-fold between testing occasions, substrate utilisation may be affected (Oosthuyse & Bosch, 2010). Systematic differences in PFO and FAT_MAX_ were assessed by a mixed-design ANOVA (within-subject: Trial A and B; between-subject: group) and dependent-sample *t*-tests were conducted with Bonferroni adjusted p-values applied to control for multiple comparisons and for when significant main or interaction effects were detected.

There was no significant main effect of time (*p* = 0.063) or group (*p* = 0.233), but a significant time*group interaction effect (*p* = 0.004). However, post-hoc tests revealed no significant differences. A follow-up one-way ANOVA performed on the difference in PFO between Trial A and Trial B found a significant main effect of group. This showed that the difference in PFO in females whose oestradiol (but not progesterone concentrations) were two-fold greater in one trial compared to the other, was larger compared to females whose oestradiol and progesterone concentrations were less than two-fold between trials (*p* = 0.005). No other significant effects were evident between these groups. Moreover, dependent sample *t*-tests found no systematic bias in PFO across any groups when using Bonferroni adjusted *p* values (> 0.0125). This noted, as the difference in estimates of PFO from Trial A and Trial B approached statistical (*p* = 0.016) in the females whose oestradiol (but not progesterone concentrations) were two-fold greater in one trial compared to the other, in addition to visual inspection indicating a potential systematic difference between Trial A and Trial B (Supplementary fig. 1a), sensitivity analysis was performed with and without these five female participants when analysing the day-to-day reliability of PFO across groups in the reproductive status and contraception use sub-group analysis. As highlighted in the main manuscript, exclusion of these five participants did not affect the outcome or interpretation of the data, so they were included in all analyses.

Additionally, as among female participants differences in concentrations of oestradiol and progesterone from Trial A to Trial B were not consistently in the same direction, the above statistical analysis was performed also on data when differences in concentrations were in the same direction (this involved swapping Trial data in all but the group where oestradiol and progesterone concentrations were below a two-fold difference between Trial A and Trial B). In this analysis, a significant main effect of time was apparent (*p* = 0.042). Post-hoc tests revealed that a higher PFO was estimated in Trial A (0.29 g·min^-1^) compared to Trial B (0.26 g·min^-1^ *p* = 0.042). However, neither a significant main effect of group (*p* = 0.233) nor trial*group interaction effect (*p* = 0.641) was apparent. Similarly, a one-way ANOVA revealed no main effect of group on the difference in PFO between Trial A and Trial B (*p* = 0.641). Additionally, dependent sample *t*-tests with Bonferroni adjusted p values revealed no systematic bias in any group (*p* values = 0.0125). Furthermore, visual inspection of individual data shows variability in both directions (Supplementary fig. 1c). Collectively, the above indicates that in this study changes in oestradiol concentrations above two-fold between Trial A and Trial B do not appear to impact estimates of PFO.

**FAT_MAX_**

There was no significant main effect of time (*p* = 0.111), group (*p* = 0.650) nor a significant time*group interaction effect (*p* = 0.592) for FAT_MAX_. Similarly, no significant main effect of group on the difference in FAT_MAX_ between Trial A and Trial B was apparent (*p* = 0.592). Dependent sample *t*-tests also found no significant systematic bias across groups (*p* values > 0.190). Furthermore, visual inspection of individual data shows variability in both directions (Supplementary fig. 1b)

As per above, further statistical analysis was also conducted so differences in concentrations of oestradiol and progesterone between Trial A and Trial B were consistently in the same direction. In this analysis, no main effect of time (*p* = 0.799), group (*p* = 0.649), nor time*group interaction effect (*p* = 0.525). This was consistent with the one-way ANOVA that found no main effect of group on the difference in FAT_MAX_ between Trial A and Trial B (*p* = 0.525) and the dependent sample *t*-tests that found no systematic bias across any of the groups (*p* values > 0.314). Furthermore, visual inspection of individual data shows variability in both directions (Supplementary fig. 1d). In summary, the above indicates that in this study changes in oestradiol concentrations above two-fold between Trial A and Trial B do not appear to impact estimates of FAT_MAX_.

**Supplementary tables**

**Supplementary table 1.** Female reproductive status and contraceptive use

|  |  | Oestradiol  (pmol⋅L^-1^) | | Progesterone  (nmol⋅L^-1^) | |
| --- | --- | --- | --- | --- | --- |
|  | N | A | B | A | B |
| Eumenorrheic | 24 | - | - | - | - |
| Matched | 10 | 153.0 (344.0) | 241.8 (661.3) | 0.68  (3.23) | 0.57  (3.86) |
| Non-Matched* | 7 | 472.9 (482.7) | 319.4 (1039.6) | 37.49 (70.0) | 4.04  (65.4) |
| Unknown | 7 | - | - | - | - |
| Contraception | 22 | 99.2 (1148.6) | 118.4 (2504.6) | 0.65  (8.22) | 0.75 (27.07) |
| Combined pill | 10 | 18.4  (78.1) | 18.4 (116.8) | 0.52  (0.85) | 0.66  (0.52) |
| Progesterone-only pill | 4 | 285.6 (713.9)^‡^ | 118.4 (177.6) | 0.59  (0.34)^‡^ | 0.62  (3.90) |
| IUD | 5 | 95.8 (1067.8)^‡‡^ | 81.3 (2458.4) | 0.65 (4.89)^‡‡^ | 0.64  (10.95) |
| IUS | 3 | 95.8  (523.8) | 91.5  (273.1) | 0.66  (7.3) | 0.65  (26.4) |
| Post-menopausal | 3 | 18.4  (0)^‡‡‡^ | 18.4 (0) | 0.44  (0.12)^‡‡‡^ | 0.38  (0.15) |

Data presented as median (range) unless otherwise stated. A = Trial A; B = Trial B; Non-matched, *n* = 6 for blood analysis due to *n* = 1 had no metabolic data. Unknown = reported to be eumenorrheic, but a blood sample was not obtained on either or both Trial A and Trial B. Combined pill, *n* = 6 for blood analysis as blood sample was not obtained on either or both Trial A and Trial B. Progesterone only pill, ^‡^*n* = 4 for blood analysis as a blood sample was not obtained on Trial A for *n* = 1. IUD, ^‡‡^*n* = 4 for blood sample analysis as a blood sample was not obtained on Trial A for *n* = 1. Post-menopausal, ^‡‡‡^*n* = 2 for blood analysis as a blood sample was not obtained on Trial A for *n* = 1.

**Supplementary Table 2.** Day-to-day reliability of the different data analysis approaches applied to determine peak fat oxidation and FAT_MAX_

| **Peak fat oxidation (g⋅min^−1^; *n* = 72)** | | | | | | | | | |
| --- | --- | --- | --- | --- | --- | --- | --- | --- | --- |
|  | MV | | | P2 | | | SIN | | |
|  | A | B | Avg | A | B | Avg | A | B | Avg |
| Mean ± SD | 0.32  ± 0.10 | 0.32  ± 0.11 | 0.32  ± 0.10 | 0.31  ± 0.10 | 0.30  ± 0.10 | 0.30  ± 0.10^***^ | 0.32  ± 0.11 | 0.31  ± 0.11 | 0.31  ± 0.10 |
| Bias ± SD (g⋅min^−1^) | 0.00 ± 0.08 | | | - 0.01 ± 0.08 | | | -0.01 ± 0.09 | | |
| *r* | 0.71 (0.57 – 0.81 | | | 0.71 (0.57 – 0.81) | | | 0.67 (0.51 – 0.78) | | |
| CV (%) | 19 (16 – 22) | | | 20 (17 – 23) | | | 21(18 – 24) | | |
| TE (g⋅min^−1^) | 0.06 (0.05 – 0.07) | | | 0.06 (0.05 – 0.07) | | | 0.06 (0.05 – 0.07) | | |
| 95% LoA  (±; g⋅min^−1^) | 0.16 | | | 0.15 | | | 0.17 | | |
| **FAT_MAX_ (%V̇O_2_peak; (*n* = 72)** | | | | | | | | | |
| Mean ×/÷ SD | 37  ×/÷ 1.36 | 37  ×/÷ 1.41 | 37  ×/÷ 1.31^§*^ | 39  ×/÷ 1.2^§*^ | 39  ×/÷ 1.21 | 39  ×/÷ 1.20 | 36  ×/÷ 1.28 | 36  ×/÷ 1.31 | 36  ×/÷ 1.25^§***^ |
| Bias ratio | 1.01 | | | 1.02 | | | 1.00 | | |
| *r* | 0.41 (0.23 – 0.56) | | | 0.70 (0.59 – 0.79) | | | 0.53 (0.37 – 0.66) | | |
| CV (%) | 29 (25 – 34) | | | 11 (10 – 13) | | | 20 (17– 23) | | |
| TE ratio | 1.28 (1.25 – 1.34) | | | 1.11 (1.10 – 1.13) | | | 1.20 (1.17 – 1.23) | | |
| 95% ratio LoA (×/÷) | 2.00 | | | 1.34 | | | 1.64 | | |

Data presented as mean (± 95% CI) unless otherwise stated; FAT_MAX_ data is transformed and presented as mean (×/÷ 95% CI) unless otherwise stated; *n* = 34 and 38 females and males, respectively; A = Trial A; B = Trial B; SD = standard deviation; Avg = Average of Trial A and B (mean ± SD); *r* = Pearson correlation; CV = within-subject coefficient of variation; TE = typical error; LoA = limits of agreement; FAT_MAX_ LoA = SD Ratios; *** *p* < .001, P2 *vs* MV and SIN. ^§*^ *p* < .05 MV *vs* P2; ^§***^ *p* < .001 SIN *vs* P2.

**Supplementary Table 3.** Day-to-day reliability of peak fat oxidation and FAT_MAX_ split by biological sex

| **Peak Fat Oxidation (g⋅min^−1^; *n* = 50 and 47)** | | | | | | | | | | |
| --- | --- | --- | --- | --- | --- | --- | --- | --- | --- | --- |
|  | Males | | | | | Females | | | | |
|  | A | | B | Avg | | A | | B | | Avg |
| Mean ± SD | 0.36  ± 0.12 | | 0.34  ± 0.13 | 0.35  ± 0.12*** | | 0.28  ± 0.10 | | 0.28  ± 0.10 | | 0.28  ± 0.09*** |
| Bias ± (g⋅min^−1^) | -0.02 ± 0.09 | | | | | 0.00 ± 0.07 | | | | |
| *R* | 0.73 (0.56 – 0.84) | | | | | 0.74 (0.57 – 0.85) | | | | |
| CV (%) | 22 (17 – 25) | | | | | 20 (15 – 24) | | | | |
| TE (g⋅min^−1^) | 0.07 (0.06 – 0.08) | | | | | 0.05 (0.04 – 0.06) | | | | |
| 95% LoA  (±; g⋅min^−1^) | 0.19 | | | | | 0.14 | | | | |
| **FAT_MAX_ (%V̇O_2_peak; *n* = 50 and 47)** | | | | | | | | | | |
| Mean ×/÷ SD | 36  ×/÷ 1.41 | 35  ×/÷ 1.45 | | | 35  ×/÷ 1.35 | 38  ×/÷ 1.27 | 40  ×/÷ 1.28 | | 39  ×/÷ 1.23 | |
| Bias ratio | 0.97 | | | | | 1.03 | | | | |
| *R* | 0.44 (0.23 – 0.61) | | | | | 0.43 (0.21 – 0.61) | | | | |
| CV (%) | 31 (26 – 38) | | | | | 20 (17 – 25) | | | | |
| TE ratio | 1.31 (1.26 – 1.38) | | | | | 1.20 (1.17 – 1.25) | | | | |
| 95% ratio LoA (×/÷) | 2.10 | | | | | 1.66 | | | | |

Data presented as mean (± 95% CI) unless otherwise stated; FAT_MAX_ data is transformed and presented as mean (×/÷ 95% CI) unless otherwise stated; A = Trial A; B = Trial B; SD = standard deviation; Avg = Average of Trial A and B (mean ± SD); r = Pearson correlation; CV = within-subject coefficient of variation; TE = typical error; LoA = limits of agreement; FAT_MAX_ LoA = SD Ratios; ****p* < 0.001.

**Supplementary Table 4.** Day-to-day reliability of peak fat oxidation and FAT_MAX_ split by cardiorespiratory fitness

| **Peak Fat Oxidation (g⋅min^−1^; *n* = 54 and 41)** | | | | | | |  |
| --- | --- | --- | --- | --- | --- | --- | --- |
|  | Untrained | | | Trained | | | |
|  | A | B | Avg | A | B | Avg | |
| Mean ± SD | 0.28  ± 0.09 | 0.27  ± 0.08 | 0.27  ± 0.08*** | 0.35  ± 0.11 | 0.34  ± 0.12 | 0.34  ± 0.10 *** | |
| Bias ± SD (g⋅min^−1^) | -0.01 ± 0.07 | | | -0.01 ± 0.10 | | | |
| *R* | 0.70 (0.53 – 0.82) | | | 0.61 (0.37 – 0.77) | | | |
| CV (%) | 21 (16 – 25) | | | 21 (17 – 25) | | | |
| TE (g⋅min^−1^) | 0.05 (0.04 – 0.06) | | | 0.07 (0.06 – 0.09) | | | |
| 95% LoA (±; g⋅min^−1^) | 0.14 | | | 0.20 | | | |
| **FAT_MAX_ (%V̇O_2_peak; *n* = 54 and 41)** | | | | | | |  |
| Mean ×/÷ SD | 36  ×/÷ 1.3 | 35  ×/÷ 1.32 | 35  ×/÷ 1.27 | 38  ×/÷ 1.39 | 40  ×/÷ 1.43 | 39  ×/÷ 1.32 | |
| Bias ratio | 0.96 | | | 1.05 | | | |
| *R* | 0.55 (0.38 – 0.69) | | | 0.32 (0.07 – 0.54) | | | |
| CV (%) | 20 (17.1 – 24.3) | | | 33.0 (27.3 – 41.9) | | | |
| TE ratio | 1.20 (1.17 – 1.24) | | | 1.33 (1.27 – 1.42) | | | |
| 95% ratio LoA (×/÷) | 1.66 | | | 2.20 | | | |

Data presented as mean (± 95% CI) unless otherwise stated; FAT_MAX_ is transformed and data presented as mean (×/÷ 95% CI) unless otherwise stated; A = Trial A; B = Trial B; SD = standard deviation; Avg = Average of Trial A and B (mean ± SD); r = Pearson correlation; CV = within-subject coefficient of variation; TE = typical error; LoA = limits of agreement; FAT_MAX_ LoA = SD Ratios; *** *p* ≤ .001 Untrained *vs* trained.;

**Supplementary table 5.** Day-to-day reliability of peak fat oxidation and FAT_MAX_ split by fat mass index classification

| **Peak Fat Oxidation (g⋅min^−1^; *n* = 26, 58 and 12)** | | | | | | | | | |
| --- | --- | --- | --- | --- | --- | --- | --- | --- | --- |
|  | Fat deficient | | | *Healthy* | | | Excess adiposity | | |
|  | A | B | Avg | A | B | Avg | A | B | Avg |
| Mean ± SD | 0.38  ± 0.15 | 0.38  ± 0.16 | 0.38  ± 0.15*** | 0.29  ± 0.09 | 0.28  ± 0.09 | 0.29  ± 0.08*** | 0.31  ± 0.10 | 0.30  ± 0.07 | 0.30  ± 0.08 |
| Bias ± SD (g⋅min^−1^) | 0.01 ± 0.11 | | | -0.02 ± 0.08 | | | -0.02 ± 0.06 | | |
| *R* | 0.76 (0.52 – 0.89) | | | 0.66 (0.48 – 0.78) | | | 0.81 (0.44 – 0.94) | | |
| CV (%) | 23 (16 -28) | | | 21 (17 -25) | | | 15 (11 – 18) | | |
| TE (g⋅min^−1^) | 0.08 (0.06 – 0.11) | | | 0.05 (0.05 – 0.07) | | | 0.04 (0.03 – 0.07) | | |
| 95% LoA  (±; g⋅min^−1^) | 0.22 | | | 0.15 | | | 0.12 | | |
| **FAT_MAX_ (%V̇O_2_peak; *n* = 26, 58 and 12)** | | | | | | | | | |
| Mean ± SD | 42  ± 13 | 46  ± 14 | 44  ± 11*** | 37  ± 11 | 36  ± 11 | 37  ± 10*** | 40  ± 8 | 40  ± 12 | 40  ± 8 |
| Bias ± SD (%V̇O_2_peak) | 4 ± 16 | | | 1 ± 11 | | | 0 ± 13 | | |
| *R* | 0.32 (-0.08 – 0.63) | | | 0.49 (0.26 – 0.66) | | | 0.19 (-0.43 – 0.69) | | |
| CV (%) | 26 (19 – 32) | | | 20 (14 – 25) | | | 22 (11 – 29) | | |
| TE (%V̇O_2_peak) | 11 (9 – 16) | | | 8 (7 – 10) | | | 9 (6 – 15) | | |
| 95% LoA  (±; %V̇O_2_peak) | 31 | | | 23 | | | 25 | | |

Data presented as mean (± 95% CI) unless otherwise stated; A = Trial A; B = Trial B; SD = standard deviation; Avg = Average of Trial A and B (mean ± SD); r = Pearson correlation; CV = within-subject coefficient of variation; TE = typical error; LoA = limits of agreement; Obese excluded *n* = 1; *** *p* ≤ .001, Fat deficient *vs healthy*.

**Supplementary table 6.** Day-to-day reliability of peak fat oxidation and FAT_MAX_ split by habitual physical activity levels

| **Peak Fat Oxidation (g⋅min^−1^; *n* = 27, 39 and 25)** | | | | | | | | | |  |
| --- | --- | --- | --- | --- | --- | --- | --- | --- | --- | --- |
|  | Lightly active | | | Active | | | Very active | | | |
|  | A | B | Avg | A | B | Avg | A | B | Avg | |
| Mean ± SD | 0.28  ± 0.10 | 0.26  ± 0.07 | 0.27  ± 0.08** | 0.32  ± 0.10 | 0.30  ± 0.12 | 0.31  ± 0.10 | 0.38  ± 0.14 | 0.37  ± 0.16 | 0.37  ± 0.14** | |
| Bias ± SD (g⋅min^−1^) | -0.02 ± 0.07 | | | -0.02 ± 0.08 | | | -0.01 ± 0.11 | | | |
| *r* | 0.73 (0.48 – 0.87) | | | 0.75 (0.58 - 0.86) | | | 0.74 (0.49 - 0.88) | | | |
| CV (%) | 19 (12 – 25) | | | 20 (14 – 24) | | | 21 (15 – 26) | | | |
| TE (g⋅min^−1^) | 0.05 (0.04 – 0.06) | | | 0.05 (0.04 – 0.07) | | | 0.08 (0.06 – 0.11) | | | |
| 95% LoA  (±; g⋅min^−1^) | 0.13 | | | 0.15 | | | 0.21 | | | |
| **FAT_MAX_ (%V̇O_2_peak; *n* = 27, 39 and 25)** | | | | | | | | | |  |
| Mean ×/÷ SD | 37 ×/÷ 1.21 | 34 ×/÷ 1.24 | 35 ×/÷ 1.20 | 37 ×/÷ 1.40 | 36 ×/÷ 1.45 | 37 ×/÷ 1.35 | 39 ×/÷ 1.41 | 42 ×/÷ 1.38 | 41 ×/÷ 1.32 | |
| Bias ratio | 0.92 | | | 0.98 | | | 1.08 | | | |
| *r* | 0.57 (0.30 – 0.75) | | | 0.47 (0.24 – 0.66) | | | 0.43 | | | |
| CV (%) | 15 (12 – 20) | | | 29 (24 – 37) | | | 29 (23 – 40) | | | |
| TE ratio | 1.15 (1.12 – 1.19) | | | 1.29 (1.24 – 1.37) | | | 1.29 (1.23 – 1.39) | | | |
| 95% ratio LoA (×/÷) | 1.46 | | | 2.04 | | | 2.01 | | | |

Data presented as mean (± 95% CI) unless otherwise stated; FAT_MAX_ data is transformed and presented as mean (×/÷ 95% CI) unless otherwise stated; deviation; A = Trial A; B = Trial B; SD = standard deviation; Avg = Average of Trial A and B (mean ± SD); *r* = Pearson correlation; CV = within-subject coefficient of variation; TE = typical error; LoA = limits of agreement; FAT_MAX_ LoA = SD Ratios; ***p* < 0.01, Lightly active *vs* very active.

**Supplementary table 7.** Day-to-day reliability of peak fat oxidation and FAT_MAX_ split by menstrual cycle status and contraceptive use

| **Peak Fat Oxidation (g⋅min^−1^; *n* = 10, 6, 10 and 6)** | | | | | | | | | | | | |
| --- | --- | --- | --- | --- | --- | --- | --- | --- | --- | --- | --- | --- |
|  | Matched | | | Non-Matched | | | Contraceptive use – Combined Pill | | | Unknown | | |
|  | A | B | Avg | A | B | Avg | A | B | Avg | A | B | Avg |
| Mean ± SD | 0.25  ± 0.10 | 0.26  ± 0.10 | 0.26  ± 0.09 | 0.28  ± 0.09 | 0.27  ± 0.06 | 0.27  ± 0.07 | 0.31  ± 0.05 | 0.30  ± 0.06 | 0.31 ± 0.05 | 0.27  ± 0.09 | 0.24  ± 0.08 | 0.25  ± 0.08 |
| Bias ± SD (g⋅min^−1^) | 0.02 ± 0.10 | | | -0.01 ± 0.08 | | | 0.01 ± 0.06 | | | -0.03 ± 0.05 | | |
| *R* | 0.52 (-0.17 – 0.86) | | | 0.48 (-0.54 – 0.93) | | | 0.42 (-0.29 – 0.83) | | | 0.81 (-0.01 – 0.98) | | |
| CV (%) | 32 (19 – 41) | | | 19 (9 – 25) | | | 14 (7 - 18) | | | 13 (0 – 19) | | |
| TE (g⋅min^−1^) | 0.07 (0.05- 0.13) | | | 0.06 (0.04 – 0.14) | | | 0.04 (0.03 – 0.08) | | | 0.04 (0.02 – 0.09) | | |
| 95% LoA  (±; g⋅min^−1^) | 0.19 | | | 0.16 | | | 0.12 | | | 0.11 | | |
| **FAT_MAX_ (%V̇O_2_peak; *n* = 10, 6, 22 and 6 )** | | | | | | | | | | | | |
| Mean ± SD | 38  ± 10 | 37  ± 11 | 38  ± 10 | 39  ± 10 | 48  ± 13 | 44  ± 10 | 43 ± 13 | 42 ± 6 | 42 ± 7 | 37  ± 5 | 35  ± 2 | 36  ± 4 |
| Bias ± SD (%V̇O_2_peak) | -1 ± 7 | | | 10 ± 12 | | | -1 ± 15 | | | -2 ± 3 | | |
| *r* | 0.78 (-0.29 – 0.94) | | | 0.55 (-0.47 – 0.94) | | | -0.13 (-0.70 – 0.55) | | | 0.99 (0.93 – 1.00) | | |
| CV (%) | 13 (6 – 17) | | | 21 (0 – 35) | | | 23 (12 – 30) | | | 7 (0 – 9) | | |
| TE (%V̇O_2_peak) | 5 (4 – 10) | | | 8 (5 – 20) | | | 11 (7 – 19) | | | 2 (1 – 6) | | |
| 95% LoA (±; %V̇O_2_peak) | 14 | | | 23 | | | 29 | | | 7 | | |

Data presented as mean (± 95% CI) unless otherwise stated; A = Trial A; B = Trial B; SD = standard deviation; Avg = Average of Trial A and B (mean ± SD); r = Pearson correlation; CV = within-subject coefficient of variation; TE = typical error; LoA = limits of agreement.

**Supplementary figure legends**

**Supplementary figure 1a – 1d.** Comparison of peak fat oxidation (g⋅min^-1^) and FAT_MAX_ (%V̇O_2_peak) between Trial A and Trial B to observe whether differences in concentrations of oestradiol and progesterone between trials affects estimates of PFO and FAT_MAX_. Supplementary fig. 1a and 1b reflect data independent of whether the differences in oestradiol and progesterone concentrations were in the same direction across trials, whereas Supplementary fig. 1c and 1d. display swapped data i.e. differences in oestradiol and progesterone concentrations between Trial A and B were in the same direction for all participants. ≥ 2 E2 < 2 P = Oestradiol and progesterone concentrations were ≥ and < two-fold between Trial A and B, respectively. < 2 E2 & P = Oestradiol and progesterone concentrations were both < two-fold between Trial A and B. ≥ 2 E2 & P = Oestradiol and progesterone concentrations were both ≥ two-fold between Trial A and B. < 2 E2 ≥ 2 P = Oestradiol and progesterone concentrations were < and ≥ two-fold between Trial A and B, respectively. The solid thick line represents mean ± SD with individual data denoted by the thin lines. Measured values approach used to determine PFO and FAT_MAX_.

**Supplementary figure 2a – 2h.** Comparison of peak fat oxidation (g⋅min^-1^) between Trial A and B for the following sub-group analyses: Fig 2a – 2c depict the different data analysis approaches applied to determine PFO (measured values [2a], construction of a polynomial second order [P2; 2b] and the SINE model [SIN; 2c]); Sex (2d); Cardiorespiratory fitness classifications (2e); Fat mass index (2f); Habitual physical activity levels (2g) and menstrual cycle status and contraceptive use (2h). The solid thick line represents mean ± SD with individual data denoted by the thin lines. Measured values approach used to determine PFO and FAT_MAX_ for Supplementary fig. 2d – 2h.

**Supplementary figure 3a – 3h.** Comparison of FAT_MAX_ (%V̇O_2_peak) between Trial A and B for the following sub-group analyses: Fig 3a – 3c depict the different data analysis approaches applied to determine FAT_MAX_ (measured values [3a], construction of a polynomial second order [P2; 3b] and the SINE model [SIN; 3c]); Sex (3d); Cardiorespiratory fitness classifications (3e); Fat mass index (3f); Habitual physical activity levels (3g) and menstrual cycle status and contraceptive use (3h). The solid thick line represents mean ×/÷ SD with individual data denoted by the thin lines. Measured values approach used to determine PFO and FAT_MAX_ for Supplementary fig. 3d – 3h.

**References**

Oosthuyse, T., & Bosch, A. N. (2010). The effect of the menstrual cycle on exercise metabolism: implications for exercise performance in eumenorrhoeic women. *Sports Med, 40*(3), 207-227. doi:10.2165/11317090-000000000-00000
