# Supplementary figures and images for: The day-to-day reliability of peak fat oxidation and FATMAX
Source: Eur J Appl Physiol. 2020 Jun 1;120(8):1745–59. doi: 10.1007/s00421-020-04397-3 (PMC7340634; doi:10.1007/s00421-020-04397-3)

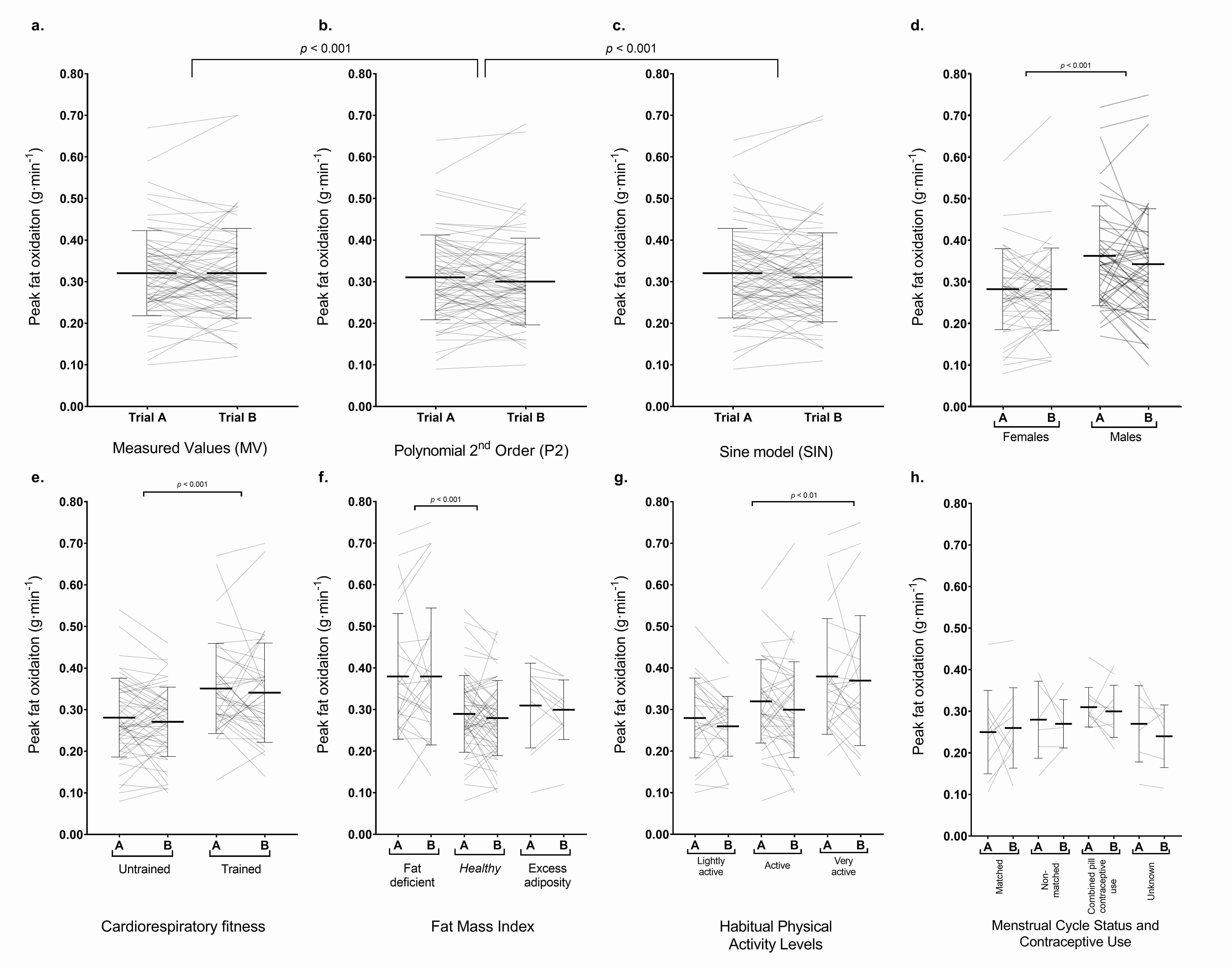

Supplement: Supplementary file 2 — Supplementary file2 (JPG 833 kb) [file 421_2020_4397_MOESM2_ESM.jpg]

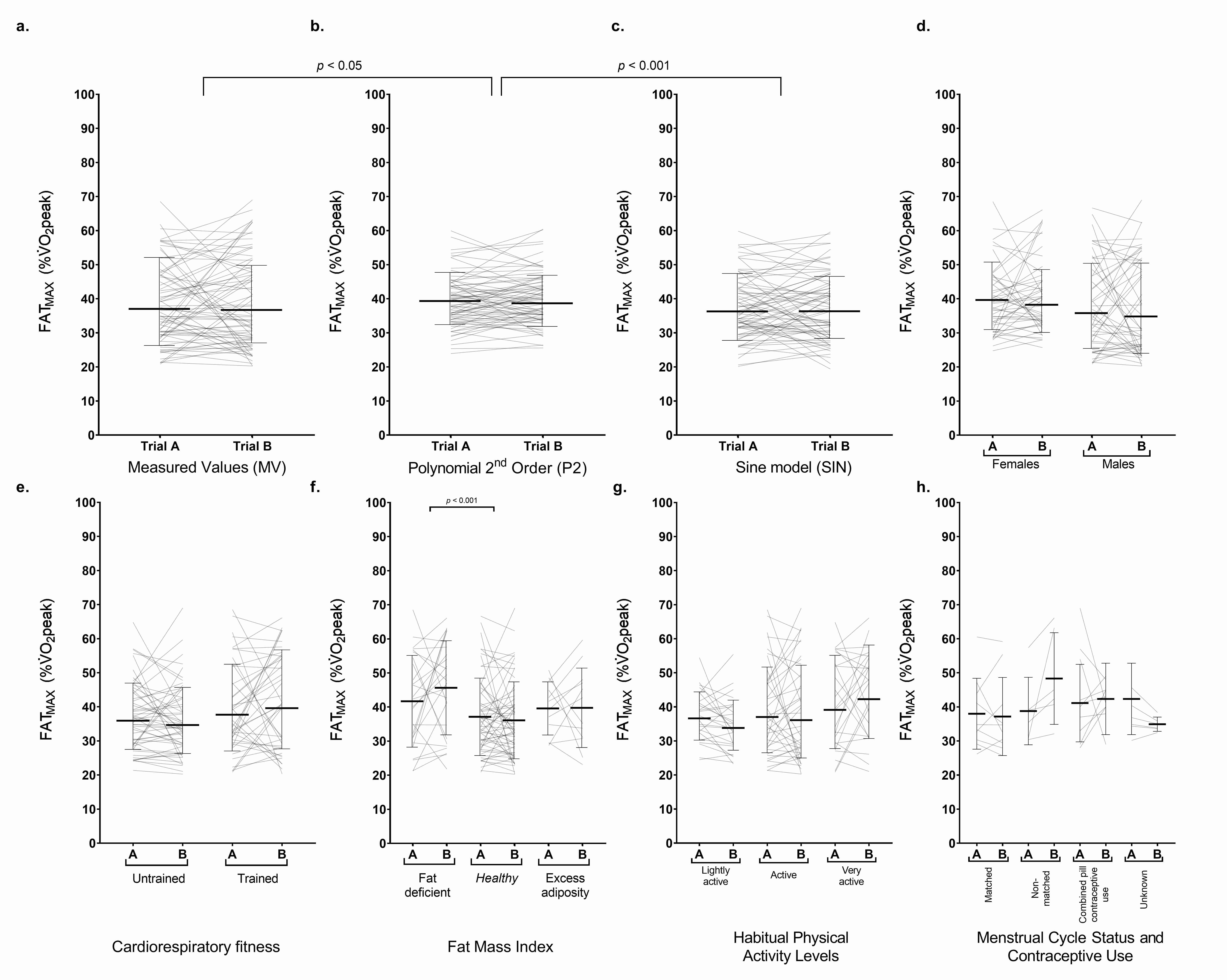

Supplement: Supplementary file 3 — Supplementary file3 (JPG 797 kb) [file 421_2020_4397_MOESM3_ESM.jpg]
